# Dengue Virus 1 in Buenos Aires from 1999 to 2010: Towards Local Spread

**DOI:** 10.1371/journal.pone.0111017

**Published:** 2014-10-24

**Authors:** Estefanía Tittarelli, Alicia S. Mistchenko, Paola R. Barrero

**Affiliations:** 1 Laboratorio de Virología, Hospital de Niños “Ricardo Gutiérrez”, Ciudad Autónoma de Buenos Aires, Buenos Aires, Argentina; 2 Consejo Nacional de Investigaciones Científicas y Técnicas (CONICET), Buenos Aires, Argentina; 3 Comisión de Investigaciones Científicas de la Provincia de Buenos Aires (CIC), Buenos Aires, Argentina; University of Missouri, United States of America

## Abstract

Dengue virus (DENV) is a public health problem representing the most important arthropod-borne viral disease in humans. In Argentina, Northern provinces have reported autochthonous cases since 1997, though these outbreaks have originated in bordering countries, where co-circulation of more than one serotype has been reported. In the last decade, imported dengue cases have been reported in Buenos Aires, the urban area of Argentina with the highest population density. In 2009, a dengue outbreak affected Buenos Aires and, for the first time, local transmission was detected. All cases of this outbreak were caused by DENV-1. In this report, we present the full-length sequences of 27 DENV-1 isolates, corresponding to imported cases of 1999–2000, as well as local and imported cases of the 2009 and 2010 outbreaks. We analyzed their phylogenetic and phylodynamic relationships and their global and local spread. Additionally, we characterized their genomic and phenotypic features. All cases belonged to DENV-1 genotype V. The most recent ancestor for this genotype was dated ∼1934, whereas that for the 2009 outbreak was dated ∼2007. The mean rates of nucleotide substitution were 4.98E-4 and 8.53E-4 subs./site/yr, respectively. We inferred an introduction from Paraguay in 1999–2000 and mainly from Venezuela during 2009–2010. Overall, the number of synonymous substitutions per synonymous site significantly exceeded the number of non-synonymous substitutions per site and 12 positively selected sites were detected. These analyses could contribute to a better understanding regarding spread and evolution of this pathogen in the Southern Cone of South America.

## Introduction

Dengue viruses (DENV) have a single-stranded RNA genome and belong to the genus Flavivirus, family *Flaviviridae*. DENV are classified into four serotypes, DENV-1-4, which are further divided into different genotypes. Dengue poses a public health problem. Infections cause a wide range of clinical manifestations, ranging from mild fever to severe disease, including hemorrhagic fever and dengue shock syndrome. DENV are transmitted among humans by mosquitoes, of the species *Aedes aegypti and Aedes albopictus,* and more than 100 countries have reported endemic transmission [1].

Although only nine countries, mostly located in Southeast Asia, had severe dengue before 1970, dengue transmission is currently more common in urban and semi-urban areas worldwide. In the Americas, a significant increase in dengue incidence has been observed in the last two decades [2]. Almost all countries bordering Argentina have reported dengue cases, with co-circulation of more than one serotype. DENV-1 has been reported in Brazil since 1986, with annual outbreaks since 1995; and in Bolivia since 1987, with annual outbreaks since 1999. Paraguay has also reported DENV-1 since 1988, with outbreaks during 1999–2002 and since 2009, whereas Chile has reported DENV-1 since 2002. No DENV cases have been reported in Uruguay [3], [4].

In Argentina, most cases concentrate in areas close to Northern borders, in contact with neighboring countries but seldom in its capital city, Buenos Aires, where the first outbreak with local transmission was detected in 2009. Metropolitan Buenos Aires is a densely populated area (more than 12 million inhabitants) being one of the biggest cities of South America, located in the Southern part of the continent (latitude 34°S). Metropolitan Buenos Aires has established an emergent pathogens monitoring program by the end of the 1990s. Before the 2009 outbreak, only cases acquired in the Northern provinces of Argentina or in foreign countries were reported.

During 1999, while an outbreak of DENV-1 was reported in Paraguay, the first imported cases were detected in Metropolitan Buenos Aires without evidence of local transmission [5], [6]. Ten years later, between October 2008 and June 2009, an outbreak of DENV-1 was reported in Bolivia and Northern Argentine provinces, with more than 25,000 cases in Argentina. This outbreak reached Metropolitan Buenos Aires where the highest number of DENV-1 cases was reported between February and May 2009 and local transmission was detected for the first time [7]. DENV-1 circulation was also reported in the following summer (2010) in Metropolitan Buenos Aires, without sustained local transmission.

Describing the genetic structure and population dynamics of DENV is crucial to determine the underlying evolutionary processes [8]–[10]. Accordingly, we obtained the full-length sequences of 27 DENV-1 isolates from 1999–2000, 2009 and 2010. We analyzed their phylogenetic and phylodynamic relationships, and their global and local spread to contribute to better understand the evolution of this pathogen. Additionally, we characterized their genomic features, and evaluated growth characteristics of the isolates in cell culture.

## Materials and Methods

### 1. Patients samples

Between 1999 and 2010, plasma or serum samples from 336 patients with confirmed DENV-1 infection were studied in our laboratory. Seventy-two viruses were isolated and amplified in cell culture. Among them, 27 isolates were randomly chosen to represent the early isolates (1999–2000, corresponding to imported cases) and the recent ones (2009–2010, which included local transmission) (Table 1).

**Table 1:** Data of the DENV-1 strains isolated and sequenced in this study.

Isolate	GenBankAccesionNumber	Collectiondate	Epidemiologicalweek	Daysafterfever	Age	Sex	C6/36passage	Sourcecountry	Residentiallocation
HNRG20	AY277664	May 16, 1999	20	7	48	F	2	Paraguay	−34.699955; −58.392140
HNRG28	AY277665	March 15, 2000	11	4	47	M	1	Brazil, center	−34.677589; −58.561691
HNRG44	AY277659	April 10, 2000	15	2	39	M	3	Paraguay	−34.733149; −58.460999
HNRG48	AY277666	April 12, 2000	15	5	27	F	1	Paraguay	−34.642618; −58.410576
HNRG12188	KC692495	February 3, 2009	5	7	13	F	1	Bolivia, Sta.Cruz de la Sierra	−34.678679; −58.467028
HNRG12447	KC692496	February 19, 2009	7	1	28	F	1	Argentina,Salta	−34.625298; −58.555584
HNRG12560	KC692497	March 2, 2009	9	2	21	M	1	Argentina,Catamarca	−34.715916; −58.544849
HNRG12589	KC692498	March 3, 2009	9	3	10	M	1	Argentina,Catamarca	−34.823048; −58.437066
HNRG13154	KC692499	March 31, 2009	13	4	64	M	2	Argentina,MetropolitanBuenos Aires	−34.646456; −58.540291
HNRG13188	KC692500	April 1, 2009	13	4	40	M	1	Argentina,Salta	−34.664801; −58.546340
HNRG13301	KC692501	April 6, 2009	14	4	63	M	1	Argentina,Chaco	−27.066667; −61.066667
HNRG13405	KC692502	April 9, 2009	14	4	17	M	2	Argentina,MetropolitanBuenos Aires	−34.655772; −58.503177
HNRG13561	KC692503	April 14, 2009	15	4	58	M	1	Argentina,Jujuy	−34.626009; −58.378804
HNRG13707	KC692504	April 16, 2009	15	1	73	M	1	Argentina,MetropolitanBuenos Aires	−34.599147; −58.434278
HNRG13708	KC692505	April 16, 2009	15	3	35	M	1	Argentina,Sgo. del Estero	−34.598812; −58.456632
HNRG14043	KC692506	April 22, 2009	16	4	23	F	2	Argentina,MetropolitanBuenos AIres	−34.632682; −58.477134
HNRG14076	KC692507	April 23, 2009	16	4	39	M	2	Argentina,MetropolitanBuenos AIres	−34.715485; −58.541904
HNRG14194	KC692508	April 24, 2009	16	5	62	M	2	Argentina,MetropolitanBuenos AIres	−34.630909; −58.540485
HNRG14635	KC692509	May 5, 2009	18	1	68	M	1	Argentina,MetropolitanBuenos AIres	−34.654153; −58.509092
HNRG15417	KC692510	May 28, 2009	21	3	67	F	1	Argentina,MetropolitanBuenos Aires	−34.623056; −58.523442
HNRG24827	KC692511	February 10, 2010	6	3	47	F	1	Brazil, north	−34.583984; −58.462960
HNRG25001	KC692512	February 17, 2010	7	4	8	M	2	Argentina,Misiones	−34.683333; −58.466667
HNRG27213	KC692513	April 26, 2010	17	1	56	M	1	Brazil, north	−34.586963; −58.415014
HNRG27486	KC692514	May 3, 2010	18	4	47	F	1	Argentina,MetropolitanBuenos Aires	−34.704079; −58.320599
HNRG28226	KC692515	May 14, 2010	19	4	39	M	1	Argentina,Corrientes	−34.61681; −58.429734
HNRG28425	KC692516	May 18, 2010	20	5	63	M	1	Venezuela	−34.579147; −58.477911
HNRG37945	KC692517	December 29, 2010	52	4	43	F	2	Peru	−34.616822; −58.399887

Residential location is expressed as latitude; longitude.

#### 1.1 Isolation in cell culture

Serum or plasma from DENV-1-positive samples were used as inoculum for *Aedes albopictus* C6/36 cells (ATCC CRL-1660). Cells were harvested at days 6–7 of infection, and infections confirmed by indirect immunofluorescence with the monoclonal antibody Anti-Dengue Virus-1 MAB10227 (EMD Millipore Corporation, Billerica, MA, USA). Viral stocks were obtained with less than 3 passages in cell culture. Viral titers were assessed in plaque assays and recorded as plaque forming units/mL (PFU/mL). Briefly, viral suspension stocks were serially 10-fold diluted in MEM Eagle medium with 2% fetal bovine serum (FBS). Two hundred microliters were inoculated on cell monolayers in a 24-well plate. After 1 h of virus adsorption at 37°C, cells were overlayed with 3% of carboxymethylcellulose–MEM medium containing 2% FBS. After incubation at 37°C for 7 days, they were stained with crystal violet-formaldehyde, dried at room temperature and plaques were counted.

### 2. Genome amplification, analysis and annotation

Cell-free supernatants were used to obtain genomic viral RNA by using PureLink Viral RNA/DNA Mini Kit (Invitrogen, Carlsbad, CA, USA). Overlapping RT-PCR products were obtained for the viral genome as described previously [6], [9]. Sequences were analyzed in an ABI3500 genetic analyzer and consensus sequences were obtained by compiling 4–6 overlapping reads with SeqScape Software v2.7 (Applied Biosystems, Foster City, CA, USA). Amino acid sequences were inferred using the universal code and polymorphisms were expressed by comparing the sequences with the reference sequence FGA/89 (AF226687.2).

Viral genomes were annotated online by Genome Annotation Transfer Utility (GATU) through the Virus Pathogen Database and Analysis Resource (ViPR) [11], [12]. The sequences were submitted to GenBank (KC692495–KC692517). In addition, samples from 1999–2000 were re-sequenced and GenBank records (AY277659-64-65-66) were updated.

To determine the natural selection acting on the viruses analyzed in our laboratory, the ratio of non-synonymous (dN) to synonymous (dS) substitutions per site (dN/dS) was estimated at every codon in the alignment by Datamonkey web server and at the overall alignment (ω = dN/dS) by DNAsp v.5 (DNA Sequence Polymorphism) [13], [14]. The Datamonkey analysis was performed using the following codon-based maximum likelihood (ML) methods: SLAC [single likelihood ancestor counting], FEL [fixed effects likelihood], REL [random effects likelihood], and MEME [mixed effects model of evolution] at the specified significance levels (p<0.1 and Bayes factor >50) [15]. N-glycosylation sites were predicted by NetNGlyc server [16].

### 3. Genotyping

In order to genotype our sequences, phylogenetic inferences were evaluated by ML (PhyML v.20120412 software [17]) and Bayesian criteria (MrBayes software v.3.2.1 [18]). Initial random trees were used to infer the ML tree and the branch support was evaluated by non-parametric bootstrapping with 100 pseudo-replica. The convergence of the Monte Carlo Markov Chains (MCMC) implemented in the Bayesian criteria was evaluated in TRACER v.1.5 with an effective sample size (ESS) >200; the initial 10% of the run length was discarded as burn-in. Consensus trees were visualized with FigTree v.1.4.0. The total number of sequences included in this analysis consisted of 77 DENV full-length sequences including 27 sequences obtained in our laboratory and 50 representative reference sequences from DENV-1-4 and their respective genotypes retrieved from the NCBI Dengue Virus Resource [19] and NIAD ViPR [12] (Table S1). Sequences were aligned with MUSCLE [20]. The appropriate nucleotide substitution model for the sequence alignment was selected using jModelTest v 0.1.1 [21].

### 4. Phylodynamic and phylogeographic analyses

To evaluate the global evolution and spread of DENV-1 genotype V sequences, a discrete phylogeographic analysis was performed. The sequences included in this dataset consisted of 50 DENV full-length sequences including 27 sequences obtained in our laboratory and 23 representative reference sequences from DENV-1 genotype V [12], [19]. Each sequence was associated to the original source country and the year of collection.

In order to characterize the 2009 DENV-1 genotype V outbreak in Metropolitan Buenos Aires a continuous phylogeographic analysis was performed. The sequences included in this dataset consisted of 2009 DENV full-length sequences obtained in our laboratory. These sequences were associated to the residential latitude and longitude and the date of collection (yyyy-mm-dd).

Bayesian coalescent-based methods implemented in the BEAST package [22] were used to estimate the most recent common ancestor (tMRCA), the mean rate of nucleotide substitution, the demographic histories and the phylogeographic analyses. All BEAST run logs were analyzed with TRACER after evaluating the convergence of the MCMC as described in the previous section. The history of infections with DENV-1 genotype V was reconstructed using Bayesian Skyline Plots (BSPs) implemented in TRACER [23]. Ninety-percent highest probability densities were considered (95% HPD). The most clade credibility tree (MCCT) was visualized by TreeAnnotator v.1.7.5. The phylogeographic analysis was summarized with SPREAD (Spatial Phylogenetic Reconstruction of Evolutionary Dynamics) v.1.0.6 [24] and the diffusion process was visualized with Google Earth (Google Inc., Mountain View, CA, USA). All the models used in BEAST package were selected by Bayes factor.

### 5. Plaque area assay

To evaluate the plaque area morphology, confluent cultures of BHK-21 cells in 6-well plates were infected with DENV at a multiplicity of infection (MOI) of 0.001 for 1 h at 37°C, washed 3 times, overlaid with 3% carboxymethyl-cellulose and incubated at 37°C for 6 days. Then, viral plaques were fixed with formaldehyde, stained with crystal violet and visualized, counted and measured in a Gel DOC XR^+^ system, with Quantity One v4.6.8 software (Bio-Rad). The area was evaluated in duplicate.

### 6. Biological properties of DENV-1 isolates

To assess the biological behavior of isolates, seven of the 27 isolates were randomly chosen to represent isolates from 1999–2000 (HNRG48/00); isolates from 2009 (HNRG12447/09, 13154/09, 14043/09 and 14635/09), and isolates from 2010 (HNRG24827/10 and 25001/10).

Confluent cultures of BHK-21 cells in 24-well plates were infected in quadruplicate with DENV at a MOI of 0.001. Three different aliquots of the cell-free supernatants were harvested at 1, 2, 3, 4, 5 and 6 days post-infection to evaluate the kinetics of viral replication (as RNA copy number and plaque assay) and the production of the viral nonstructural protein 1 (NS1).

#### 6.1 Kinetics

**i)** Viral RNA copy number was evaluated by reverse transcription followed by real-time PCR (qPCR) (adapted from [25]). Briefly, the capsid region was amplified using the primer pair 5′-nt255-ATACCYCCAACAGCAGGAATT-nt275-3′ and 5′-nt403-AGCATRAGGAGCATGGTCAC-nt384-3′, and the probe 5′-nt276-6FAM-TTGGCTAGATGGRGCTCATTCAAGAAGAAT-TAMRA-nt305-3′. Reverse transcription was performed at 50°C for 30 min followed by 5 min at 95°C to inactivate iScript Reverse Transcriptase, 45 cycles of 95°C for 30 sec, and 30 sec at 55°C with iTaq DNA polymerase (Bio-Rad). The qPCRs were performed in duplicate on a CFX-96 real-time PCR detection system (Bio-Rad). Negative and positive controls were included in each qPCR assay. The limit of detection was 60 copy/µL. Copy number quantification was performed by a standard curve obtained from 10-fold dilutions of the cloned quantified synthetic positive controls, obtained by cloning amplicon of the DENV-1 Hawaii strain into the pGEM-T vector (Promega Inc. Madison, WI, USA). Data were collected with Bio-Rad CFX Manager Software (v2.1.1022.0523) and reported following the Minimum Information for Publication of Quantitative Real-Time PCR Experiments (MIQE) guidelines [26].

**ii)** Plaque assay was performed after infecting the BHK-21 monolayer with the cell free supernatants collected each day as described above for viral titration by plaque assay.

#### 6.2. NS1 protein

NS1 is a highly conserved, multi-functional glycoprotein, which is strongly immunogenic. The NS1 protein is secreted as an hexamer and the amount secreted is closely correlated to the titer of DENV [27]–[29]. NS1 was detected in culture supernatants collected each day with an antigen capture enzyme-linked immunosorbent assay NS1 Platelia kit (BioRad, Hercules, CA, USA). Semi-quantitative results are expressed as absorbance at 450 nm/cut-off value (Sample Ratio = SR), according to manufacturer’s instructions.

### 7. Statistical analyses

Statistical analyses were performed using Wilcoxon test or t-test and one-way analysis of variance complemented by Tukey test of the software package InfoStat (v. 2012) [30]. Statistical significance was defined as p<0.05.

### 8. Ethics statement

The study was revised and approved by the Medical Ethic and Research Committees of “Ricardo Gutiérrez” Children’s Hospital, Buenos Aires, Argentina (IRB N° 10.46). Informed consent was not obtained, as patient information was anonymized and de-identified prior to analysis.

## Results

### 1. Genetic analysis

**Figure 1:**
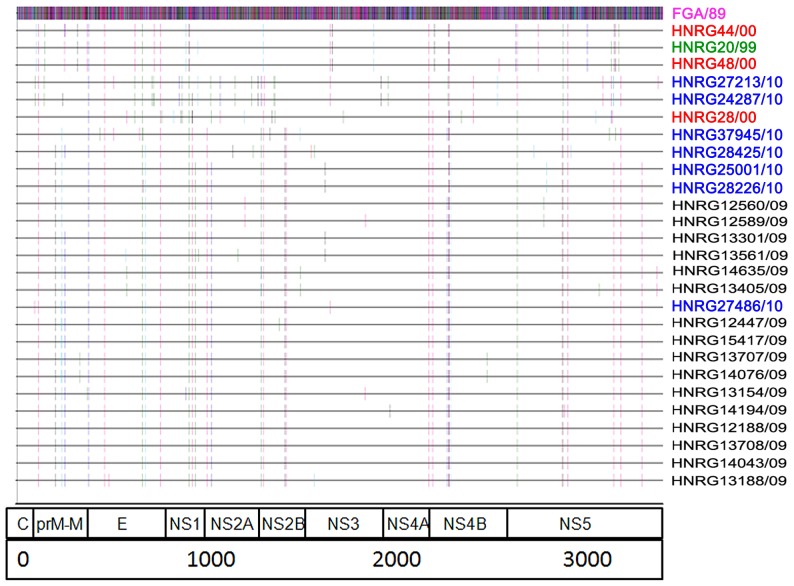
Location of non-synonymous changes in the polyprotein of DENV-1 strains in relation to the reference sequence FGA/89. Polar and non-polar amino acids are coded as follows: hydrophobic in pink (Ala, Phe, Ile, Leu, Met, Pro, Val, Trp); polar but uncharged in light blue (Cys, Gly, Asn, Gln, Ser, Thr, Tyr); negatively charged in gray (Asp, Glu); and positively charged in red (His, Lys, Arg).

We sequenced a total of 10,735 nucleotides per isolate of transcribed RNA. Compared to the DENV-1 genotype V strain FGA/89, a total of 1,057 sites were mutated, with 919 synonymous changes and 138 non-synonymous changes. A total of 143 segregating sites were detected, 81 (56.6%) were conservative and 62 (43.4%) were non-conservative. In eight of the segregating sites, two variants were detected (Figure 1 and Table S2).

Analysis of predicted N-glycosylation sites revealed that three isolates did not conserve the 14 expected sites. HNRG28245/10 imported from Venezuela gained a glycosylation site at position NS1-1134, whereas HNRG24827/10 and 27213/10, imported from Northern Brazil, lost a glycosylation site at position NS5-3078.

Selection analysis of the sequences obtained in our laboratory was performed using the TrN nucleotide substitution model. According to at least one of the assayed methods, the dN/dS ratio evaluated at every codon revealed that 12 sites were under positive selection and 265 were under purifying selection (negatively selected), with an overall dN/dS of 0.037. Positively selected sites were located in M, NS1, NS2B, NS3, NS4B, and NS5 proteins (2, 2, 1, 3, 1, and 3, respectively) (Table S3). Also, 8 polymorphic sites were detected in E (2 non-conservative (NC)), NS1 (2, 1 NC), NS4B (1 NC), and NS5 (3 NC). Sites NS1-1068 and NS5-3122 were both positively selected and polymorphic, with 2 variants.

### 2. Genotyping, phylodynamic and phylogeographic analyses

To infer phylogenetic relationships and determine the genotype of the sequences obtained in this study, the GTR+G+I evolution model was selected. All methods applied were congruent showing the same phylogenetic relationships with high statistical support. As a result, isolates from our laboratory belonged to DENV-1 genotype V (Figure S1).

**Figure 2:**
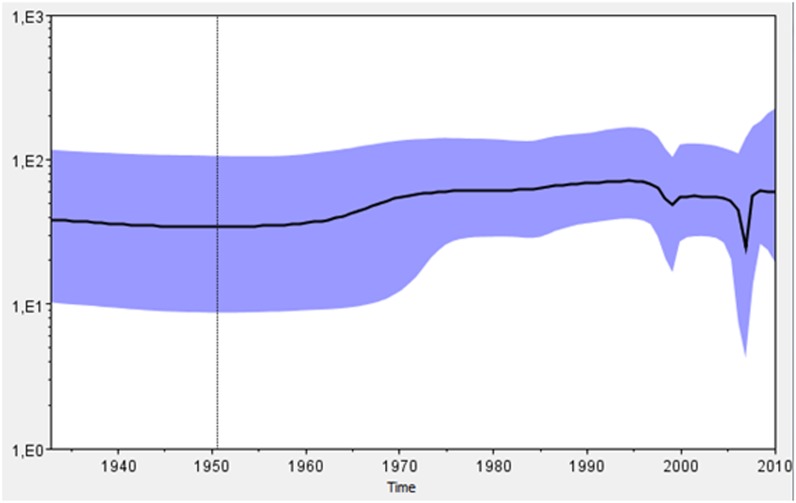
Bayesian skyline plot for DENV-1 genotype V. The population size of DENV-1 genotype V (Y axis) vs time in years (X axis) is shown. Ninety-percent highest probability densities are considered.

**Figure 3:**
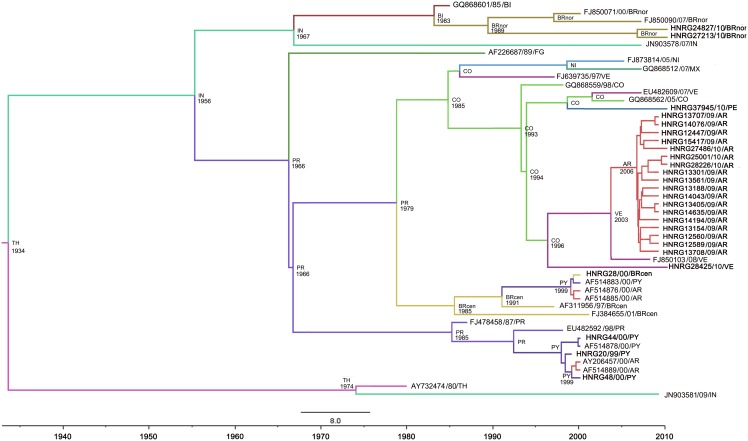
Most clade credibility tree obtained by a discrete phylogeographic analysis of DENV-1 genotype V. The probable country location and age of the ancestors are shown on the nodes (5E+8 generations sampling every 5E+4). Sequences included in the analysis are noted as GenBank accession number/collection date/source country for sequences downloaded from GenBank, and as HNRGnumber/collection date/source country for sequences obtained in our laboratory. Abbreviations of the countries are as follows: AR: Argentina, BI: British Virgin Islands, BRcen: Center of Brazil, BRnor: North of Brazil, CO: Colombia, FG: French Guiana, IN: India, MX: Mexico, NI: Nicaragua, PE: Peru, PR: Puerto Rico (USA), PY: Paraguay, TH: Thailand, VE: Venezuela.

**Figure 4:**
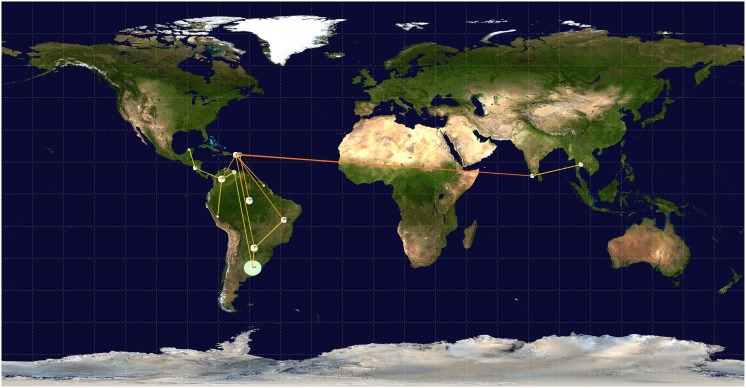
Inferred spread obtained by a discrete phylogeographic analysis of DENV-1 genotype V. The KML file for visualization in Google Earth is available as a supplementary dataset (Video S1).

Viral phylodynamics and phylogeographic reconstructions were evaluated for genotype V under the TIM2+I+G evolution model. The best demographic and clock models were Coalescent Bayesian Skyline and Lognormal relaxed clock (uncorrelated), respectively. Coalescent analyses showed that the most recent ancestor of DENV-1 genotype V was dated approximately 76 years ago (∼1934) (95% HPD 58.40–94.95). Since then, the population has had a steady size until a first decay inferred around 1998. A deeper second decay was inferred around 2007 (Figure 2). The mean rate of nucleotide substitution was 4.9821E-4 subs./site/yr (95% HPD 3.98E-4-5.97E-4). We found an initial spread from Thailand to India in two waves: the first around 1956, with extensive geographical spread (95% HPD 1944–1965), and a second one without further spread. We inferred an introduction of the virus into the Americas, specifically to Puerto Rico and British Virgin Islands around 1966 (95% HPD 1958–1974) and 1983 (95% HPD 1981–1984), respectively. From these two Caribbean countries, the virus spread to different Latin American countries, such as Colombia, Northern and Center of Brazil, and Paraguay, with the highest state probabilities (SP) (0.28, 0.68, 0.27 and 0.89 respectively). After reaching Colombia, the virus spread to Peru, Nicaragua and diversified into different clades into Venezuela (with SP = 0.56 for the clade related to Argentina). Viruses from Nicaragua spread to Mexico and viruses from the Center of Brazil spread to Paraguay (SP = 0.62). Also, viruses from two different sources of Paraguay and Venezuela reached Argentina around 1999 (95% HPD 1999–2000) and 2006 (95% HPD 2005–2007), with SP = 0.73, 0.90 and 0.99, respectively. The discrete phylogeographic analyses are depicted by the MCCT in Figure 3, and the diffusion process is summarized in Figure 4 and Video S1.

To evaluate the temporal-spatial diffusion of the 2009 DENV-1 genotype V outbreak in Metropolitan Buenos Aires, the continuous phylogeographic analysis under the TrN+G evolution model was performed with a Brownian, Lognormal relaxed clock (uncorrelated) and Coalescent Bayesian Skyline model (5E+7 generations, sampling every 5E+3). The estimated tMRCA of these samples was 1.59 years before the last reported in our dataset (95% HPD 0.43–14.75), and dated approximately around the end of 2007. The mean rate of nucleotide substitution was 8.5313E-4 subs./site/yr (95% HPD 1.25E-6-2.42E-3). The spread rate was constant over time, with a value of 9.55 km/yr. The epicenter of the spread was located in Mataderos district, in Western Buenos Aires (Video S2).

### 3. Plaque area assay

The plaque area of 11 isolates was evaluated in duplicate. Plaque areas depicted differences that were evident even at visual inspection. To quantify the difference, the software of the Gel DOC XR^+^ was used by counting approximately 50 plaques per replicate. The mean area of recent isolates (2009–2010) from outside Metropolitan Buenos Aires (either imported or from Northern border provinces) was significantly larger than the mean area of old isolates (1999–2000) or isolates that circulated locally in 2009–2010 (0.97±0.15 mm^2^ vs 0.44±0.09 mm^2^ and 0.56±0.19 mm^2^, p = 0.005 by one-way ANOVA, p<0.01 and <0.05, respectively) (Table 2).

**Table 2:** Plaque mean area of DENV-1 isolates.

Sample	Classification	Mean area ± SD (mm^2^)
HNRG20/99	Imported	0.45±0.05
HNRG28/00	Imported	0.53±0.04
HNRG48/00	Imported	0.35±0.03
HNRG12447/09	Outside Metropolitan Buenos Aires	1.08±0.04
HNRG13154/09	Metropolitan Buenos Aires	0.45±0.05
HNRG14043/09	Metropolitan Buenos Aires	0.52±0.03
HNRG14635/09	Metropolitan Buenos Aires	0.42±0.03
HNRG24827/10	Imported	1.10±0.31
HNRG25001/10	Outside Metropolitan Buenos Aires	0.78±0.11
HNRG27486/10	Metropolitan Buenos Aires	0.84±0.01
HNRG28425/10	Imported	0.91±0.23

### 4. Biological properties of DENV-1 isolates

#### 4.1 Kinetics

DENV-1 kinetics was evaluated both by qPCR (copy/µL) and plaque assay. Since most isolates did not form plaques up to the day 4 post-infection, the plaque assay method was not suitable for a 6-day period kinetics evaluation. The best results were obtained by qPCR method which allowed the quantification from day 1 post-infection.

**Figure 5:**
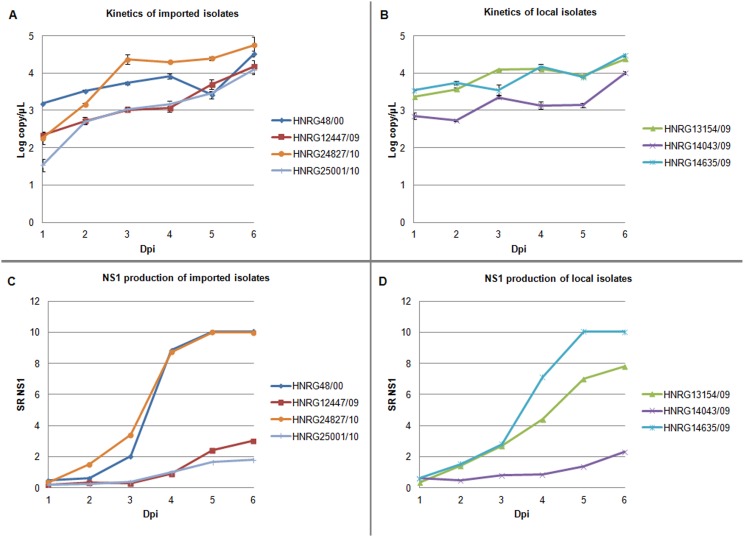
Viral RNA copies and NS1 production. Biological properties of imported (A and C) and local isolates (B and D). Viral loads are expressed as log copy/µL (A and B), points are mean values of two independent experiments. The SD are not seen in some of the points because they fall inside the symbol. NS1 production is expressed as absorbance at 450 nm/cut-off value (SR = sample ratio) (C and D). Dpi: days post-infection.

The replication rate was calculated as the mean of the curve’s slope from isolates tested. The replication rate of the 2009–2010 isolates from outside Metropolitan Buenos Aires (either imported or from Northern border provinces) was higher than that for local 2009–2010 isolates (0.41±0.06 log copy/µL/day vs 0.18±0.01 log copy/µL/day, p = 0.003, respectively). HNRG24827/10 from Brazil had the highest replication rate (0.459 log copy/µL/day) and by plaque assay showed the highest titers during the 6-day evaluation period. HNRG48/00, HNRG13154/09, and HNRG14635/09 had the lowest replication rate (0.187, 0.176, and 0.164 log copy/µL/day, respectively) (Figure 5).

#### 4.2 NS1 protein

The NS1 antigen was detected for the seven isolates since the first day of culture (Figure 5). HNRG48/00, HNRG14635/09 and HNRG24827/10 showed a significantly higher NS1 production at day 6 post-infection (SR = 10.03±0.03) than the other isolates evaluated (HNRG12447/09, HNRG13154/09, HNRG14043/09, HNRG25001/10) (SR = 3.73±2.76, p = 0.02).

## Discussion

In this study, we obtained the full-length sequence of 27 DENV-1 isolates from 1999–2000, 2009 and 2010. We analyzed their phylogenetic and phylodynamic relationships, and their global and local spread to contribute to better understand the evolution of this pathogen. Additionally, we characterized their genomic features, and evaluated growth characteristics of the isolates in cell culture.

The phylogenetic tree reconstructions confirmed that all our isolates belonged to DENV-1 genotype V, but they were from different sources. In a previous study, Chen and Vasilakis also reported that genotype V represents most DENV-1 strains collected in the Americas [31]. Most of the sequences from 1999–2000 were related to sequences retrieved from GenBank and corresponded to cases from Paraguay and the Argentine province of Misiones, consistent with the close geographical relationship between this province and the bordering country. Samples isolated in 2009 and 2010 were located at different clades and they did not form a monophyletic group.

According to the sequences of DENV-1 genotype V used for the coalescent analysis, we infer that the most recent common ancestor of all the evaluated sequences approximately dated back to 1934 (1915–1952), as reported by Villabona-Arenas [32]. The mean rate of nucleotide substitution from our global analysis resulted in 4.9821E-4 subs./site/yr (95% HPD 3.98E-4-5.97E-4). This value is in agreement with that reported by Weaver and in the same order as that previously reported for DENV-1 [10], [32]. The first entry in Latin America dated from 1966 (1959–1974) in Puerto Rico, according to previous reports [32]–[34]. With these analyses we inferred an introduction from Paraguay in 1999–2000 and later, one from Venezuela related to most samples analyzed during 2009–2010. Among other possibilities, tourism and increasing commercial relationships with Venezuela could have had a role in the introduction of the virus into Argentina.

By evaluating the demographic history of DENV-1 genotype V on the skyline plot, we observed a first decay around 1998 that might be related to the worldwide emergence of DENV-2, which also occurred in Argentina [33]. A deeper second decay was detected around 2007, which might be related to local and worldwide DENV-3 prevalence [9], [35], [36].

During 2009 DENV-1 genotype V outbreak in Metropolitan Buenos Aires, local transmission was detected for the first time. As a consequence of sustained transmission for roughly 4 months, the number of dengue cases reported in this outbreak was the highest recorded so far. The evolutionary analysis of this outbreak suggests that the common ancestor of 2009 DENV-1 genotype V outbreak in Metropolitan Buenos Aires samples dated 1.59 years before the last sample reported in our dataset (∼2007) (95% HPD 0.43–14.75). A cryptic circulation some time before the first detection by the public surveillance network [34], [36] might have been possible and it could explain the difference in time between the possible ancestor and the first case reported by the National Health system. By analyzing the geographical distribution of the patients, we found that all local cases were concentrated in a few districts in the Western part of the city, but that cases from 2010 were more disperse. The Western part of the city, an industrial area with precarious urbanization, receives much of the internal and border migrants. Mataderos district, located in the Western of the city, concentrates a total of 65,000 inhabitants and borders with the jurisdiction of La Matanza, the most populated area of Buenos Aires province (1.8 million inhabitants). La Matanza is characterized by wetlands with streams including Matanza-Riachuelo River, a basin of great importance, which could have favored the vector population growth.

Results from the genomic analysis showed punctual differences in some samples regarding glycosylation. HNRG28425/10 (imported from Venezuela) showed a mutation in the NS1 protein (gained a glycosylation site), whereas HNRG 24827/10 and 27213/10 (imported from Northern Brazil) showed a mutation in the NS5 protein (lost a glycosylation site). Although most of the sites evaluated were under purifying selection, we found 12 positively selected sites that were not present in Brazilian DENV-1 genotype V [32]. In HNRG 28/00 a positively selected site NS1 N1068S was detected. This site might be compensating the impaired replication related to the 3′UTR secondary structure reported in our previous work [6]. HNRG 24827/10 and 27213/10 samples presented the positively selected site NS5 L3122S.

The basis of DENV genetic diversity can be attributed to the RNA-dependent RNA polymerase, which does not have proof-reading activity and it is thought to produce approximately one mutation per round of genome replication [31]. In addition, the fact that some cases evaluated in this study correspond to different sources, either from other provinces or from other countries, might favor viral diversity and demonstrate the need of analyzing the selection pressures acting on these sequences. Considering the result of this analysis, we found that most codons were under negative selection and the overall dN/dS ratio was significantly lower than one (dN/dS <1). This suggests that strong purifying selection might be acting on these viruses probably due to viral protein structural constraints and the fact that the virus must be maintained in two different hosts (human and mosquito). It is worth to mention that the study was performed with low passages viruses, thus lowering the occurrence of mutations related to culture adaptation.

We found that among the 2009–2010 isolates from outside Metropolitan Buenos Aires (either imported or from Northern border provinces) the mean plaque area produced *in vitro* was significantly larger than that of older isolates (1999–2000) or isolates that circulated locally in 2009–2010. A relationship between the plaque area and the viral load was found; samples with higher replication rates during the 6-day study period had bigger areas. The case imported from Paraguay in 2000, HNRG48, showed the smallest area, whereas the case imported from Brazil in 2010, HNRG24827, showed the biggest area. Plaque size is usually related to biological properties of DENV and small size is frequently used as an attenuation marker for the development of vaccine candidates [37].

Non distinctive mutation could be associated to the high NS1 production in samples HNRG48/00, HNRG14635/09 and HNRG24827/10. This issue remains to be further analyzed.

In plaque assays most isolates did not start producing plaques before 4 days post-infection, impeding to perform kinetic studies by this method. On the other hand, viral RNA copies and NS1 production were detected since day 1 post-infection. At early times, when NS1 production is detectable, levels of mature virions are still too low to be reliably measured by plaque assay [27].

These are the first phylodynamic and phylogeographic analyses of 2009 DENV-1 genotype V outbreak in Metropolitan Buenos Aires, a non-endemic region where DENV circulation was not expected. These types of analyses in combination with a tight follow-up of the local epidemiological situation could contribute to a better understanding of DENV evolution in the Southern Cone of South America.

## Supporting Information

Figure S1 **Phylogenetic Bayesian consensus tree.** Bayesian consensus tree obtained after 9E+6 generations run in duplicate sampling every 1000 generations until convergence with an effective sample size (ESS) >200, 10% of burn-in. Trees were rooted with sylvatic DENV-4. Full-length sequences reported in this work are indicated with a black triangle.
(TIF)

Table S1 **Dataset used for phylogenetic, phylodynamic and phylogeographic analyses.**
(DOCX)

Table S2 **Replacement changes found in isolates obtained in our laboratory.**
(DOCX)

Table S3 **Positively selected sites found in the 27 full-length genomes obtained in our laboratory.**
(DOCX)

Video S1 **Interactive virtual global spread animation for DENV-1 genotype V.** The KML file can be played by Google Earth (http: earth.google.com).
(KML)

Video S2 **Interactive virtual spread animation for 2009 DENV-1 genotype V outbreak in Metropolitan Buenos Aires.** The KML file can be played by Google Earth (http: earth.google.com).
(KML)
