# Supplementary figures and images for: Dengue Virus 1 in Buenos Aires from 1999 to 2010: Towards Local Spread
Source: PLoS One. 2014 Oct 24;9(10):e111017. doi: 10.1371/journal.pone.0111017 (PMC4208802; doi:10.1371/journal.pone.0111017)

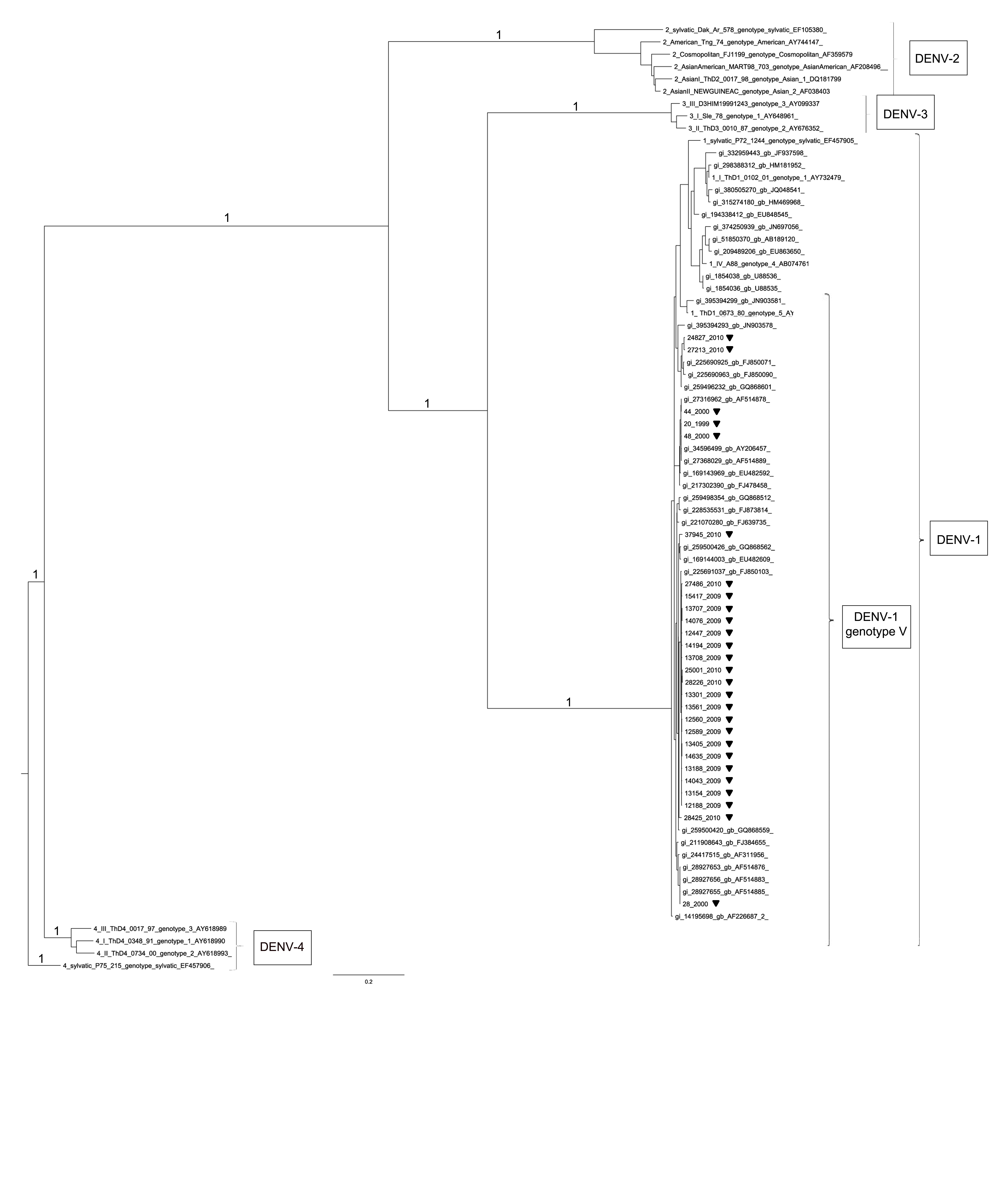

Supplement: Figure S1 — Phylogenetic Bayesian consensus tree. Bayesian consensus tree obtained after 9E+6 generations run in duplicate sampling every 1000 generations until convergence with an effective sample size (ESS) >200, 10% of burn-in. Trees were rooted with sylvatic DENV-4. Full-length sequences reported in this work are indicated with a black triangle. (TIF) [file pone.0111017.s001.tif]
