# Supplementary material for: Dengue Virus 1 in Buenos Aires from 1999 to 2010: Towards Local Spread
Source: PLoS One. 2014 Oct 24;9(10):e111017. doi: 10.1371/journal.pone.0111017 (PMC4208802; doi:10.1371/journal.pone.0111017)
Supplement: Table S1 — Dataset used for phylogenetic, phylodynamic and phylogeographic analyses. (DOCX) [file pone.0111017.s002.docx]

**Table S1. Dataset used for phylogenetic, phylodynamic and phylogeographic analyses.**

NA= not available.

* The code for samples used in BEAST package is noted. NU=not used.

**The latitude and longitude for samples used in BEAST package are noted. NU=not used.

***Full-length sequences reported in this work.
