# Supplementary material for: Dengue Virus 1 in Buenos Aires from 1999 to 2010: Towards Local Spread
Source: PLoS One. 2014 Oct 24;9(10):e111017. doi: 10.1371/journal.pone.0111017 (PMC4208802; doi:10.1371/journal.pone.0111017)
Supplement: Table S2 — Replacement changes found in isolates obtained in our laboratory. (DOCX) [file pone.0111017.s003.docx]

**Table S2. Replacement changes found in isolates obtained in our laboratory.**

*Presence of the mutation.

C=conservative amino acid change.

NC= non-conservative amino acid change.

Positively selected amino acid residues are highlighted in yellow and polymorphic sites with two variants are highlighted in grey.
