# Supplementary material for: Dengue Virus 1 in Buenos Aires from 1999 to 2010: Towards Local Spread
Source: PLoS One. 2014 Oct 24;9(10):e111017. doi: 10.1371/journal.pone.0111017 (PMC4208802; doi:10.1371/journal.pone.0111017)
Supplement: Table S3 — Positively selected sites found in the 27 full-length genomes obtained in our laboratory. (DOCX) [file pone.0111017.s004.docx]

**Table S3. Positively selected sites found in the 27 full-length genomes obtained in our laboratory.**

SLAC (single likelihood ancestor counting), FEL (fixed effects likelihood), REL (random effects likelihood) and MEME (mixed effects model of evolution). Level of significance: for SLAC, FEL and MEME p <0.1; REL posterior probability >50. Positively selected residues with significant values are shaded in violet.
